# The Superficial Anastomosing Veins of the Human Brain Cortex: A Microneurosurgical Anatomical Study

**DOI:** 10.3389/fsurg.2021.817002

**Published:** 2022-01-10

**Authors:** S. Ottavio Tomasi, Giuseppe Emmanuele Umana, Gianluca Scalia, Giuseppe Raudino, Francesca Graziano, Paolo Palmisciano, Stefano M. Priola, Pier Francesco Cappai, Crescenzo Capone, Peter M. Lawrence, Christian A. Erös, Klaus D. Martin, Bipin Chaurasia, Rosario Maugeri, Gerardo Iacopino, Valerio Da Ros, Michael T. Lawton, Christoph J. Griessenauer, Peter A. Winkler

**Affiliations:** ^1^Christian Doppler Clinic, University Hospital Salzburg, Paracelsus Medical University, Salzburg, Austria; ^2^Department of Neurosurgery, Cannizzaro Hospital, Catania, Italy; ^3^Garibaldi Hospital, Catania, Italy; ^4^Humanitas Centro Catanese di Oncologia, Catania, Italy; ^5^Division of Neurosurgery Health Sciences North, Northern Ontario School of Medicine, Sudbury, ON, Canada; ^6^G. Brotzu Hospital, Cagliari, Italy; ^7^Department of Neurosurgery, University Hospital Zürich, Zurich, Switzerland; ^8^Department of Neurosurgery, Barrow Neurological Institute (BNI), Phoenix, AZ, United States; ^9^Department of Neurosurgery, Städtisches Klinikum Dresden, Dresden, Germany; ^10^Neurochirurgie Dresden, Dresden, Germany; ^11^Department of Neurosurgery, University of Rome Tor Vergata, Rome, Italy; ^12^Department of Neurosurgery, University of Palermo, Palermo, Italy

**Keywords:** brain cortex, topography, microneurosurgery, anastomosing vein, vein of Labbé, vein of Trolard

## Abstract

**Introduction:** In this microneurosurgical and anatomical study, we characterized the superficial anastomosing veins of the human brain cortex in human specimens.

**Material and Methods:** We used 21 brain preparations fixed in formalin (5%) that showed no pathological changes and came from the autopsy sections. The superficial veins were dissected out of the arachnoid with the aid of a surgical microscope.

**Results:** We dissected nine female and 12 male brain specimens, with an average age of 71 ± 11 years (range 51–88 years). We classified the superficial veins in five types: (I) the vein of Trolard as the dominat vein; (II) the vein of Labbé as the dominant vein; (III) a dominant sylvian vein group, and the veins of Trolard and Labbé nonexistent or only rudimentary present without contact to the Sylvian vein group; (IV) very weak sylvian veins with the veins of Trolard and Labbé codominant; and V) direct connection of Trolard and Labbé bypassing the Sylvian vein group. The vein of Trolard was dominant (Type I) in 21.4% and the vein of Labbé (Type II) in 16.7%. A dominant sylvian vein group (Type III) was found in 42.9%. Type IV and Type V were found in 14.3 and 4.7% respectively.

**Conclusion:** No systematic description or numerical distribution of the superior anastomotic vein (V. Trolard) and inferior anastomotic vein (V. Labbé) has been found in the existing literature. This study aimed to fill this gap in current literature and provide data to neurosurgeons for the practical planning of surgical approaches.

## Introduction

Venous structures, especially large superficial and bridging veins, can significantly hamper neurosurgeons during the operative course. To minimize the risk of venous complications (1–10), precise knowledge of the cerebral vascular topography is critical. In this microneurosurgical and anatomical study, we characterized the superficial anastomosing veins of the human brain cortex in human specimens (11).

## Materials and Methods

We used brain preparations fixed in formalin (5%) with no pathological changes from the autopsy units of the Munich Anatomical Institute and the Pathological Institute of the Ludwig Maximilian University of Munich. Proper storage and examination of the brains were carried out in the laboratory for neurosurgical microanatomy in the Großhadern Hospital. The pachimeninges (dura mater) had been removed from all specimens. After a detailed optical inspection, the superficial veins were dissected out of the arachnoid using a surgical microscope (OPMI; Zeiss, Oberkochen, Germany); their natural course was preserved. Particular attention was paid to the confluence into other vessels.

### Anatomical Reference Structures

We selected the following anatomical structures as references for measuring the junction points of the superficial veins: central sulcus, frontomarginal sulcus, calcarine sulcus, and temporal pole. The selection was based on the following criteria: easy identification on the specimen, relative interindividual consistency of the structure, intraoperative applicability, favorable characteristics on imaging.

### Distance Measurements

**Figure 1:**
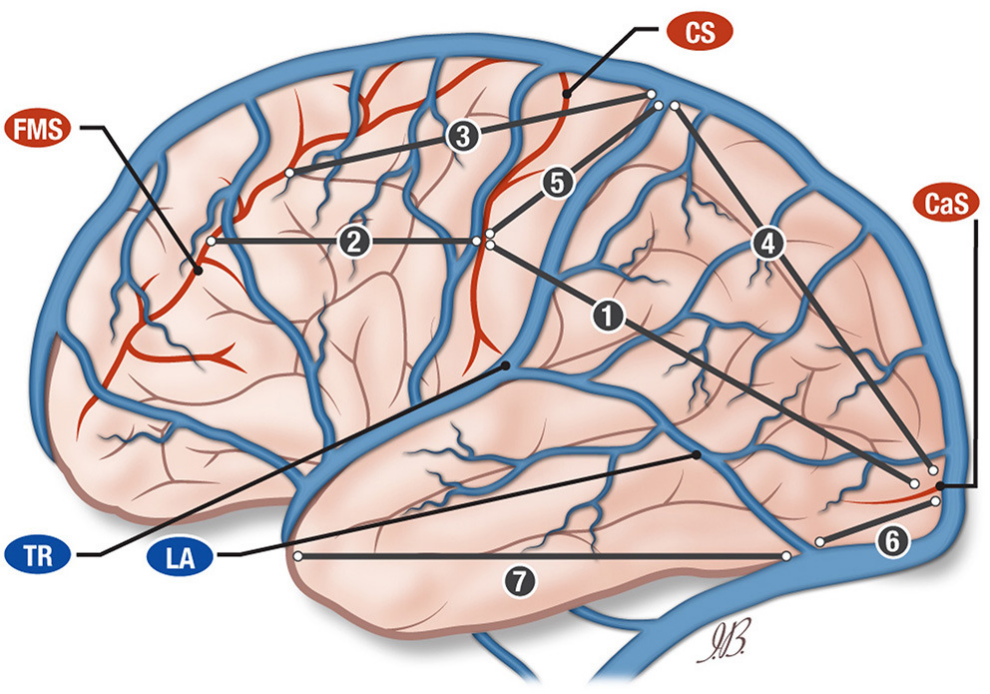
Anatomical illustration of the vein of Trolard (TR) and vein of Labbé (LA) and their relationship with central sulcus (CS), calcarine sulcus, (CAS) and frontomarginal sulcus (FMS). The drawing is illustrating the distances between central sulcus (CS), calcarine sulcus (CAS), and frontomarginal sulcus (FMS). SSS, superior sagittal sinus; CS, central sulcus; CaS, calcarine sulcus; FMS, frontomarginal sulcus; Tr, vein of Trolard; La, vein of Labbé; DTr, distance vein of Trolard-central sulcus.

We used millimeter paper to measure the distances to account for the convexity of the brain. Anatomical structures define the location as the endpoints of the measured sections. Since the size of a brain varied between individual, we also documented the location of the central sulcus with respect to the frontomarginal and calcarine sulci. We measured the following structures: central sulcus midpoint to calcarine sulcus (Figure 1, line 1); frontomarginal sulcus to central sulcus midpoint (Figure 1, line 2); frontomarginal sulcus to confluence of the vein of Trolard into the superior sagittal sinus (Figure 1, line 3); confluence of the vein of Trolard into the superior sagittal sinus to calcarine sulcus (Figure 1, line 4); central sulcus midpoint to confluence of the vein of Trolard into the superior sagittal sinus (Figure 1, line 5); calcarine sulcus to confluence of the vein of Labbé into the transverse sinus (Figure 1, line 6); confluence of the vein of Labbé into the transverse sinus to the temporal pole (Figure 1, line 7).

### Statistical Analysis

We used Microsoft Excel® for the statistical analysis. We calculated the mean, standard deviation, minimum and maximum distances for all the measurements.

## Results

The age and gender of the patients when the brains were removed were as following: nine females and 12 males, with an average age of 71 ± 11 years (range 51–88 years).

### Superficial Vein Distribution Types

We classified the superficial veins according to five types listed in Table 1. For the right hemispheres the distribution was as following: Type I: six hemispheres (14.3%); Type II: three hemispheres (7.1%); Type III: eight hemispheres (19.1%); Type IV: three hemispheres (7.1%); Type V: one hemisphere (2.4%).

**Table 1:** New classification of the large anastomosing veins the vein of Trolard, the vein of Labbé, and the Sylvian venous system.

**Type I**	V. Trolard is the dominant vein.
	The V. Labbé was not detected or showed no connection to the Sylvian veins or the V. Trolard.
**Type II**	V. Labbé is the dominant vein.
	The V. Trolard was not detected or showed no connection to the Sylvian veins or the V. Labbé.
**Type III**	The Sylvian vein group is dominant.
	V. Labbé and V. Trolard were nonexistent or only rudimentary without contact to the Sylvian vein group.
**Type IV**	All three anastomosing veins are present, V. Trolard and V. Labbé are codominant.
	The Sylvian vein group is very weak in this type, V. Trolard and V. Labbé are clearly present and communicate indirectly via the Sylvian veins.
**Type V**	Direct connection of V. Trolard and V. Labbé bypassing the Sylvian vein group.
	Both V. Trolard and V. Labbé are present, but there is no connection between these veins and the Sylvian vein group, which is also present. However, there is a direct connection between them.

For the left hemispheres the distribution was: Type I: three hemispheres (7.1%); Type II: four hemispheres (9.6%); Type III: 10 hemispheres (23.8%); Type IV: three hemispheres (7.1%); Type V: one hemisphere (2.4%).

In summary, the 42 hemispheres are classified as such: Type I: nine hemispheres (21.4%); Type II: seven hemispheres (16.7%); Type III: 18 hemispheres (42.9%); Type IV: six hemispheres (14.3%); Type V: two hemispheres (4.7%).

### Distances

#### Central Sulcus to Calcarine Sulcus and Frontomarginal Sulcus

The distance from the central sulcus midpoint to the calcarine sulcus was on average 14.67 cm (± 1.43 SD) and 14.69 cm (± 1.52 SD) on the right and the left side, respectively (Figure 1, line 1).

The distance between the central sulcus midpoint and the frontomarginal sulcus was on average 10.62 cm (± 1.46 DS) and 10.64 cm (± 1.48 SD) on the right and the left side, respectively (Figure 1, line 2).

#### Confluence of the Vein of Trolard Into the Superior Sagittal Sinus

We measured the distances from the vein of Trolard/superior sagittal sinus confluence to the frontomarginal sulcus and to the calcarine sulcus. The average of the distance between the vein of Trolard/superior sagittal sinus confluence and the frontomarginal sulcus was 11.4 cm (± 2.1 SD) and 12.2 cm (± 2.2 SD) for the right and the left side, respectively (Figure 1, line 3). The average of the distance between the vein of Trolard/superior sagittal sinus confluence and the calcarine sulcus was 14.3 cm (± 2.2 SD) and 13.4 cm (± 3.3 SD) for the right and the left side, respectively (Figure 1, line 4).

#### Central Sulcus to Calcarine Sulcus and Frontomarginal Sulcus

The average of the distances between the central sulcus midpoint and the superior sagittal sinus confluence was 0.6 cm (±2.6 SD) and 1.31 cm (±3.01 SD) for the right and the left side, respectively (Figure 1, line 5).

#### Confluence of the Vein of Labbé Into the Transverse Sinus

We measured the distances between the confluence of the vein of Labbé into the transverse sinus and the calcarine sulcus, which resulted in the following distribution: an average of 7.2 cm (± 0.9 SD) and 7.0 cm (± 1.3 SD) on the right and on the left side, respectively (Figure 1, line 6).

Calculating the distances between the confluence of the vein of Labbé into the transverse sinus and the temporal pole, we obtained an average of 9.9 cm (± 0.7 SD) and 9.7 cm (± 1.3 SD) for the right and the left side respectively (Figure 1, line 7).

The schematic representation of these measurements is showing in the Figure 1.

## Discussion

The study of the cerebral venous system, including ascending frontal veins and bridging veins, and especially the anastomotic veins, Trolard and Labbé, and the Sylvian vein, has received little attention in neurosurgery. Among the superficial veins of the cerebrum, the superior anastomotic vein (vein of Trolard), the inferior anastomotic vein (vein of Labbé), and the Sylvian veins are most at risk of occlusion since they are located along the access routes to most intracranial pathologies (2, 12). The presence of adequate anastomoses can keep venous pressure low and mitigate or even prevent the destructive effects of an occlusion (13–19).

### Superior and Inferior Anastomotic Veins: Trolard and Labbé

Regarding the presence of the veins of Trolard and Labbé, there were no significant differences between the left and the right side in the present study. Due to the lack of data on handedness, no reliable assignment to hemispheric dominance was possible. If one postulates the left hemisphere as the dominant one, there appears to be a preference for both the vein of Trolard and the vein of Labbé on that side. Such relationship did not appear in studies by either Di Chiro (20) or von Lanz (21).

### Vein of Trolard

Di Chiro (20) described that the vein of Trolard occurred more frequently in the non-dominant hemisphere. These results were obtained using angiographic imaging; in the present microanatomical study those observations could not be confirmed. Instead, we found a more frequent occurrence on the left hemisphere for both the vein of Trolard and the vein of Labbé.

In his book (21), von Lanz described the superior anastomotic vein in the area of the central sulcus or slightly dorsal to it, noting that a course toward the anterior pole of the frontal lobe can also occur. We were able to differentiate this more precisely, describing the localization of the vein of Trolard in relation to the central sulcus. When viewed from both sides, the comparison between left and right showed no significant difference, the vein of Trolard was in front of the central sulcus in 8 hemispheres and in only four cases behind it. A confluence with the central sulcus was recorded in 6 cases. When measuring the distance of the contact point with the superior sagittal sinus, it appears that the frontal span of this location is significantly larger at 6 and 5 cm on the right and left, respectively.

Stephens and Stilwell (22) outlined the veins flowing into the superior sagittal sinus into a frontal and parietal group, with no information regarding frequency and exact position. However, they note that the vein of Trolard is the most variable of all superficial veins. According to Stephens and Stilwell (22), our data show a greater variability of the vein of Trolard than the vein Labbé when the veins flow into the superior sagittal sinus or the transverse sinus.

### Vein of Labbé

Thus far, there is no clear information in the literature regarding the confluence of the vein of Labbé into the transverse sinus. A sketch in Seeger's book *Atlas of Topographical Anatomy of the Brain and Surrounding Structures* (23) shows this region, and the confluence is 10.5 cm from the temporal pole. In our measurements, we had an average of 9.9 (± 0.9) cm for the right hemisphere and 9.7 (± 1.3) cm for the left (Figure 1, line 6).

The location of the confluence of the vein of Labbé into the transverse sinus, either via the tentorium or direct, is of great importance for the planning of subtemporal and occipito-basal approaches. Like the confluence angles of the bridging veins of the cortical convexity in the direction of the superior sagittal sinus, the vein of Labbé forms a very acute angle with the temporal base or with the occipito-basal tentorium. Within this acute angle, the surgeon need to pay attention moving toward the confluence of the Vein of Labbé: if the spatula retraction is forced too much, the compression may induce venous stasis.

### A New Classification of the Large Anastomosing Veins

**Figure 2:**
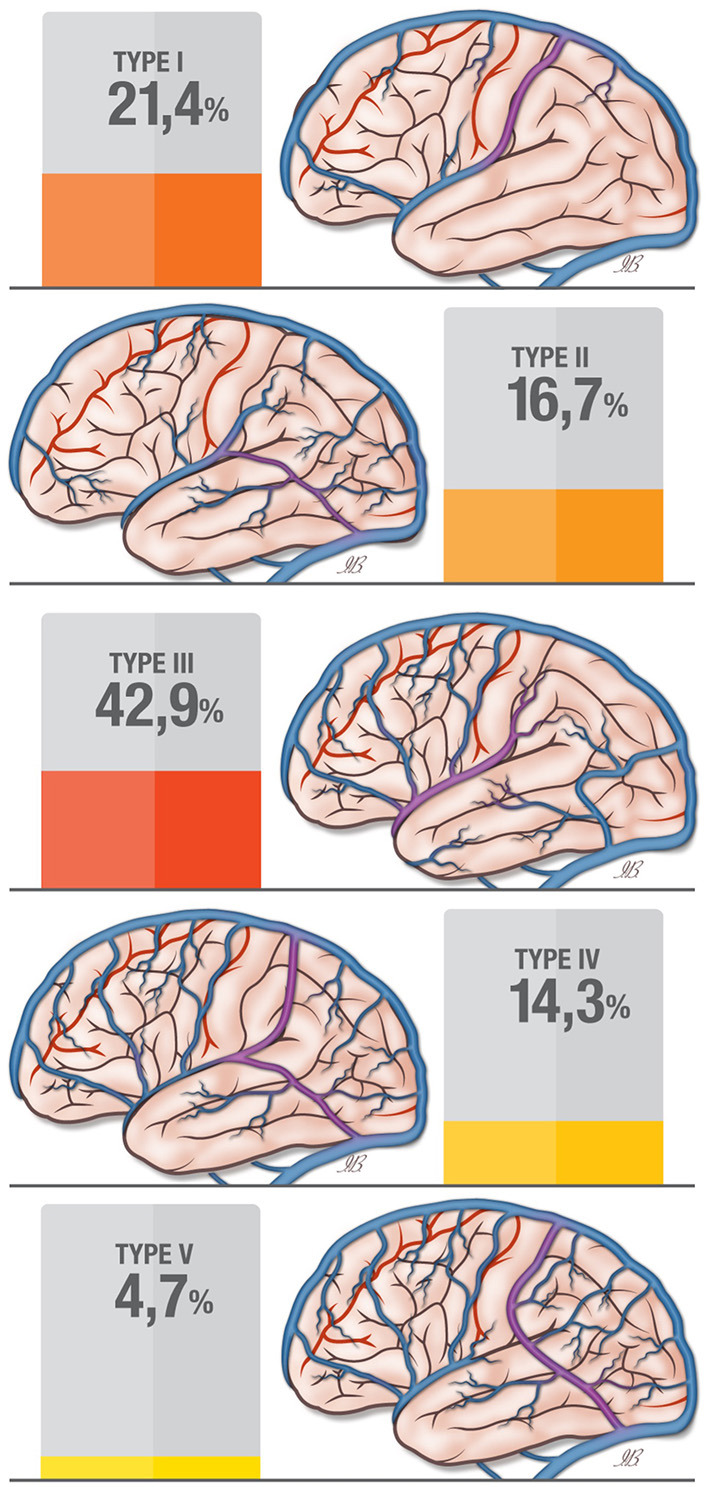
Classification for the superficial anastomosing vein patterns (according to Erös et al.) in the current study.

In a paper by Oka et al. (24), 20 cerebral hemispheres were examined for the distribution of the superficial veins. With regards to the large anastomosing veins, the vein of Trolard, the vein of Labbé, and the Sylvian venous system, the paper proposed a classification into four types: Type I: the vein of Trolard is dominant; Type II: the vein of Labbé is dominant; Type III: the Sylvian venous system is dominant; Type IV: all three anastomosing vessels are present; the veins of Trolard and of Labbé are co-dominant. The authors made no statements about the frequency of each of the types. In addition to the types described by Oka, we found another type in which there is a direct connection between the vein of Trolard and the vein of Labbé without any connection to the Sylvian venous system (Type V in Table 1 and Figure 2).

Stephens and Stilwell (22) describe significant end-to-end anastomoses between superficial veins but do not propose any precise typing. They describe that the connection of the veins of Trolard and Labbé with the Sylvian veins is already present in the fetus; these connections remain during life, but the diameter of these vessels' middle segments increases.

In the present study we performed a systematic description and a numerical distribution of the superior anastomotic vein (vein of Trolard) and inferior anastomotic vein (vein of Labbé) for the first time. Furthermore, we proposed a new classification of the large anastomotic veins, describing five types of possible venous distributions for the vein of Trolard, the vein of Labbé, and the Sylvian venous system. The first four types reflect the classification proposed by Oka et al. (24), but a fifth type, a new anatomical pattern in the variability of the superficial vein distribution, was added.

Our classification, based on the distribution of the superficial veins showed the following five types: type I with a dominant vein of Trolard, type II with a dominant vein of Labbé, type III with a dominat Sylvian vein group, type IV is characterized by the presence of all three anastomosing veins (codominant veins of Trolard and Labbé with weak a Sylvian vein), type V shows a direct connection between the vein of Trolard and the vein of Labbé without a connection with the Sylvian vein group.

## Limitation of the Study

The limitations of the study regarding the measurements are related to the use of formalin for the specimen's fixation, that could cause shrinkage of the brain, and similarly to the physiologic brain atrophy which occur in older people.

Although changes in size of the brain stem can occur. as a result of formalin fixation, changes in the cerebrum are usually smaller (25).

Therefore, these aspects do not impact or influence the main topic of the research, consisting in a new classification of the large anastomotic veins proposed in this work.

## Conclusions

The study of the superficial cerebral venous system has received little attention in neurosurgery. A thorough review of the existing literature revealed no systematic description or numerical distribution of the superior anastomotic vein (vein of Trolard) and inferior anastomotic vein (vein of Labbé). This study aims to fill this gap in the current literature and provide data to neurosurgeons for the practical planning of surgical approaches, presenting a systematic description and a numerical distribution of the superior anastomotic vein (vein of Trolard) and inferior anastomotic vein (vein of Labbé), and proposing a new classification of the large anastomotic veins. Knowledge of the drainage type present in the individual cases should be considered when planning neurosurgical operations. In addition to the optimal mental preparation for the planned resection, the exact visualization of the venous system has very high didactical value and has improved the abilities of differentiated approaches, particularly in glioma surgery, and extensive focus resections and multilobar topectomies in epilepsy surgery.

## Data Availability Statement

The original contributions presented in the study are included in the article/supplementary material, further inquiries can be directed to the corresponding author.

## Author Contributions

All authors listed have made a substantial, direct, and intellectual contribution to the work and approved it for publication.

## Conflict of Interest

The authors declare that the research was conducted in the absence of any commercial or financial relationships that could be construed as a potential conflict of interest.

## Publisher's Note

All claims expressed in this article are solely those of the authors and do not necessarily represent those of their affiliated organizations, or those of the publisher, the editors and the reviewers. Any product that may be evaluated in this article, or claim that may be made by its manufacturer, is not guaranteed or endorsed by the publisher.
